# Metabarcoding Malaise traps and soil eDNA reveals seasonal and local arthropod diversity shifts

**DOI:** 10.1038/s41598-021-89950-6

**Published:** 2021-05-18

**Authors:** Ameli Kirse, Sarah J. Bourlat, Kathrin Langen, Vera G. Fonseca

**Affiliations:** 1 Centre for Biodiversity Monitoring, Zoological Research Museum Alexander Koenig, Adenauerallee 160, 53113 Bonn, Germany; 2 Centre for Environment, Fisheries and Aquaculture Science (Cefas), Barrack Road, The Nothe, Weymouth, Dorset, DT4 8UB UK

**Keywords:** Ecology, Biodiversity, Molecular ecology

## Abstract

Forest habitats host enormous diversity, but little is known about the seasonal turnover of arthropod species between the above- and below ground forest layers. In this study, we used metabarcoding approaches to uncover arthropod diversity in different forest types and seasons. Our study shows that metabarcoding soil eDNA and Malaise trap bulk samples can provide valuable insights into the phenology and life cycles of arthropods. We found major differences in arthropod species diversity between soil samples and Malaise traps, with only 11.8% species overlap. Higher diversity levels were found in Malaise traps in summer whereas soil samples showed a diversity peak in winter, highlighting the seasonal habitat preferences and life strategies of arthropods. We conclude that collecting time series of bulk arthropod samples and eDNA in the same locations provides a more complete picture of local arthropod diversity and turnover rates and may provide valuable information on climate induced phenological shifts for long-term monitoring.

## Introduction

Forests are known to be one of the most diverse habitats on earth, providing a vast range of ecological niches, resulting in outstanding arthropod diversity^1^. Forests can be roughly divided into two habitats: the ground and the above ground layer, with both closely linked to each other by mutual relationships of the associated abiotic and biotic environment^2^. Some studies have shown how biotic interactions in soil can regulate the structure and functioning of aboveground communities. For example the presence of root-feeding invertebrates can result in differences in plant community composition and in the structure of higher above ground trophic groups^3^. The structure of forest and plant communities can be influenced by interactions in the detrital food web and because soil animals can stimulate nutrient mobilization and plant nutrient uptake, they also have the potential to indirectly affect above ground consumers^2^. While some species are present in one stratum all year round (e.g. most species of the Malacostraca, Chilopoda and Diplopoda are only present in the ground stratum), the appearance of other taxa in one or both habitats is seasonally driven. The latter is often observed for arthropods, which usually have a complex life cycle^4^, such as the large yellow underwing, *Noctua pronuba* (Lepidoptera: Noctuidae) which hibernates and pupates in the soil, before it emerges from the ground as imago in early spring^5^. The time an insect persists in any life stage varies strongly between species and often depends on season and temperature^4^. The phenology of many insects is affected by climatic changes, as already observed in wild bees and honey bees, potentially leading to mismatches in the timing of bee emergence with respect to host plant flowering^6^. When monitoring both above and below ground habitats over a longer time period, it is likely that a time lagged overlap of species occurrences can be observed between habitats, due to dispersal capabilities inherent to the various life stages of the species throughout their life cycle (e.g. larva, pupa and imago). However, depending on the target organisms, most studies are still based on a single sampling method e.g. Malaise traps, pitfall traps, light traps, bait traps or soil samples. Flying insects are often sampled with Malaise traps, but the timing and duration of sampling can strongly influence catch composition, as some species only show flight activity for a short period of time (e.g. ants)^7^. Combining several sampling strategies with time series and including various source substrates will likely both increase the number of species recovered as well as allow the recovery of a species in different life stages in the different substrates, thereby allowing the tracking and timing of emergence.

Metabarcoding environmental DNA (eDNA) studies are effective in assessing arthropod diversity in forests, either from soil^8^ or using material collected by Malaise traps^9^. The use of eDNA present in soil, coined as the “biological engine of the earth”^10^ and their invertebrate communities would be key to assess the forest belowground diversity. Environmental DNA extracted from soil samples provide reliable information on the existing diversity of several organism groups such as annelids^11^, plants^12^ and vertebrates^13,14^ but a proof of concept is still missing for several invertebrate groups, including arthropods. Insects, as well as mites, nematodes, protists and bacteria make up the bulk of terrestrial diversity and have key ecological roles in which their numbers and occurrence impact ecosystem function (e.g. pollination and forest regeneration)^15^. In this study, we hypothesised that species associated with the ground habitat in at least one life stage can be monitored with eDNA extracted from soil. However, detection of species from soil will also depend on their activity levels. While organisms that actively interact with their habitat leave a track of DNA traces (feces, excretions or epithelial cells), inactive organisms might be harder to detect unless directly captured. There are very few studies including both soil eDNA and above-ground bulk samples^8,16–18^, and only two studies have used both to address ecological questions^8,16^. Although many arthropod species are occasional inhabitants of either of the two habitats and time of occurrence is highly dependent on life stage, no study has so far addressed seasonal turnover rates between the two habitats. By monitoring both above and below ground layers over a longer time period, we propose that a time lagged overlap of species occurrences can be observed between these habitats, providing valuable data on insect phenology and in the long term, climate-associated shifts. The objective of this study was to assess diversity levels in above and below ground forest habitats but also to identify seasonal community shifts of insects and soil mesofauna using eDNA metabarcoding. We show that year-round sampling of several sample types allows for a more complete biodiversity assessment and documentation of phenological patterns in several species of flying arthropods.

## Results

### Influence of sample type on community composition

**Figure 1:**
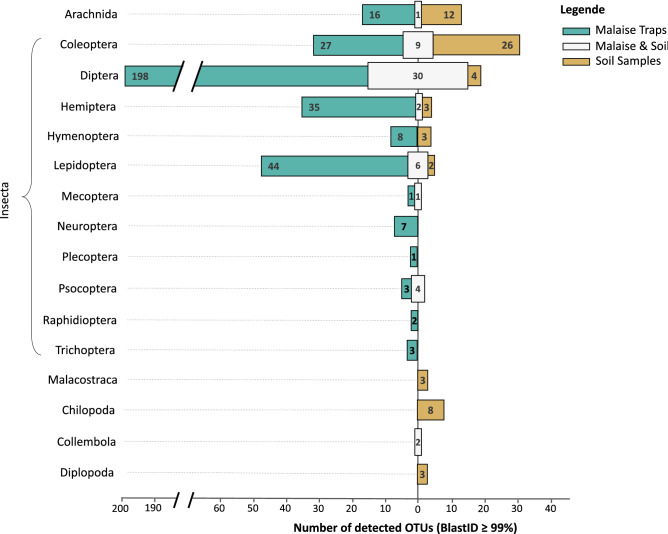
Number of unique and shared species (BlastID ≥ 99%) between Malaise traps and soil samples for the various arthropod classes found. For the class Insecta the number of shared and unique species per order is visualized.

In total, 55 arthropod species were present in both soil samples and Malaise traps, accounting for 11.8% of all arthropod species detected (464 species). A total of 74.3% of the detected arthropod species were exclusively recovered from Malaise traps (345 species), while 13.8% (64 species) were only found in the soil samples. Depending on sample type, arthropod community composition showed major differences (PERMANOVA: F_2_ = 22.057, *p* < 0.001) (Fig. 1). Data from both sample types uncovered a total of 29 arachnid species. Out of them 12 were unique to the soil samples, while 16 were exclusively recovered from the Malaise traps (Fig. 1). Only a single species was found in both sample types. All of the 14 identified species of Malacostraca, Chilopoda and Diplopoda were found only in the soil samples. In contrast, two species of Collembola were found in both sample types (Fig. 1).

The arthropod class ‘Insecta’ accounted for the highest proportion of detected species. A total of 419 insect species were found, 329 of which were exclusively recovered from the Malaise traps (78.5%) while 38 species (9.1%) were exclusively found in the soil samples. The remaining 52 species (12.4%) were detected in both sample types (Fig. 1). Because of the high functional and genetic diversity of the class Insecta, we decided to analyze this group at order level. In total, 10 insect orders were identified. The highest diversity was observed for Diptera (132 species), Coleoptera (62 species), Lepidoptera (52 species), Hemiptera (40 species) and Hymenoptera (11 species). With the exception of Coleoptera, in all insect orders, the number of species recovered from the Malaise traps exceeded those found in the soil samples (Fig. 1). Moreover, the orders Plecoptera, Neuroptera, Raphidioptera and Trichoptera were exclusively found in the Malaise traps. From the 62 Coleoptera species, 27 and 26 species were exclusively recovered from the soil samples and Malaise traps, respectively. The remaining nine species were found in both sample types. Notably, amongst the highly diverse insect orders, no overlap between the two sample types was found for the Hymenoptera. However, the number of detected hymenopteran species was exceptionally low.

### Time-lagged overlap of arthropod species occurrences between forest habitats

Malaise trap samples collected during the summer had the strongest overlap with soil samples (Supplementary Fig. 4, Table 1). Interestingly, the highest number of species was shared between Malaise trap samples from summer with soil samples of the winter season. Three species (*Athous subfuscus* (Coleoptera: Elateridae), *Corynoptera minima* (Diptera: Sciaridae), *Ctenosciara lutea* (Diptera: Sciaridae)) were recovered from the Malaise traps in summer, which were present in the soil of at least one forest type throughout the year (Table 1). Six further species were detected in the Malaise trap catches of the summer season, which were present in the soil samples of the winter season (*Malthodes fuscus* and *Malthodes mysticus* (Coleoptera; Cantharidae), *Neoitamus cyanurus* (Diptera: Asilidae), *Platypalpus nigritarsis* (Diptera: Hybotidae), *Noctua pronuba* (Lepidoptera: Noctuidae), *Epinotia tedella* (Lepidoptera: Tortricidae)) (Table 1). Additionally, three species present in the Malaise traps in the summer were detected in the soil samples during spring and winter (*Polydrusus impar* (Coleoptera: Curculionidae), *Neoitamus socius* (Diptera: Asilidae), *Neurigona quadrifasciata* (Diptera: Dolichopodidae)) and three species in the soil samples from autumn and winter (*Fannia polychaeta* (Diptera: Fanniidae), *Cydia fagiglandana* (Lepidoptera: Tortricidae), *Peripsocus subfasciatus* (Psocoptera: Peripsocidae)) (Table 1).

**Table 1:**
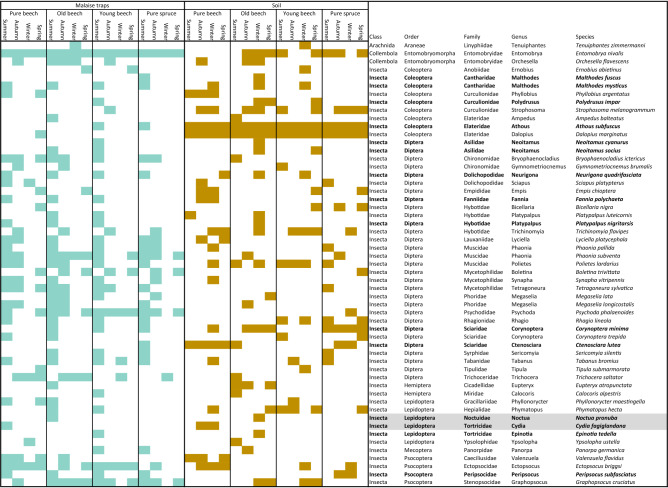
Insect species (BlastID ≥ 99%) found in either of the two study substrates soil and Malaise samples at each forest type per season.

Life cycles of the two lepidopteran species highlighted in grey are described in detail in the manuscript.

Species highlighted in bold are mentioned in the results section.

### Seasonal variation in arthropod communities depending on sample- and forest type

**Figure 2:**
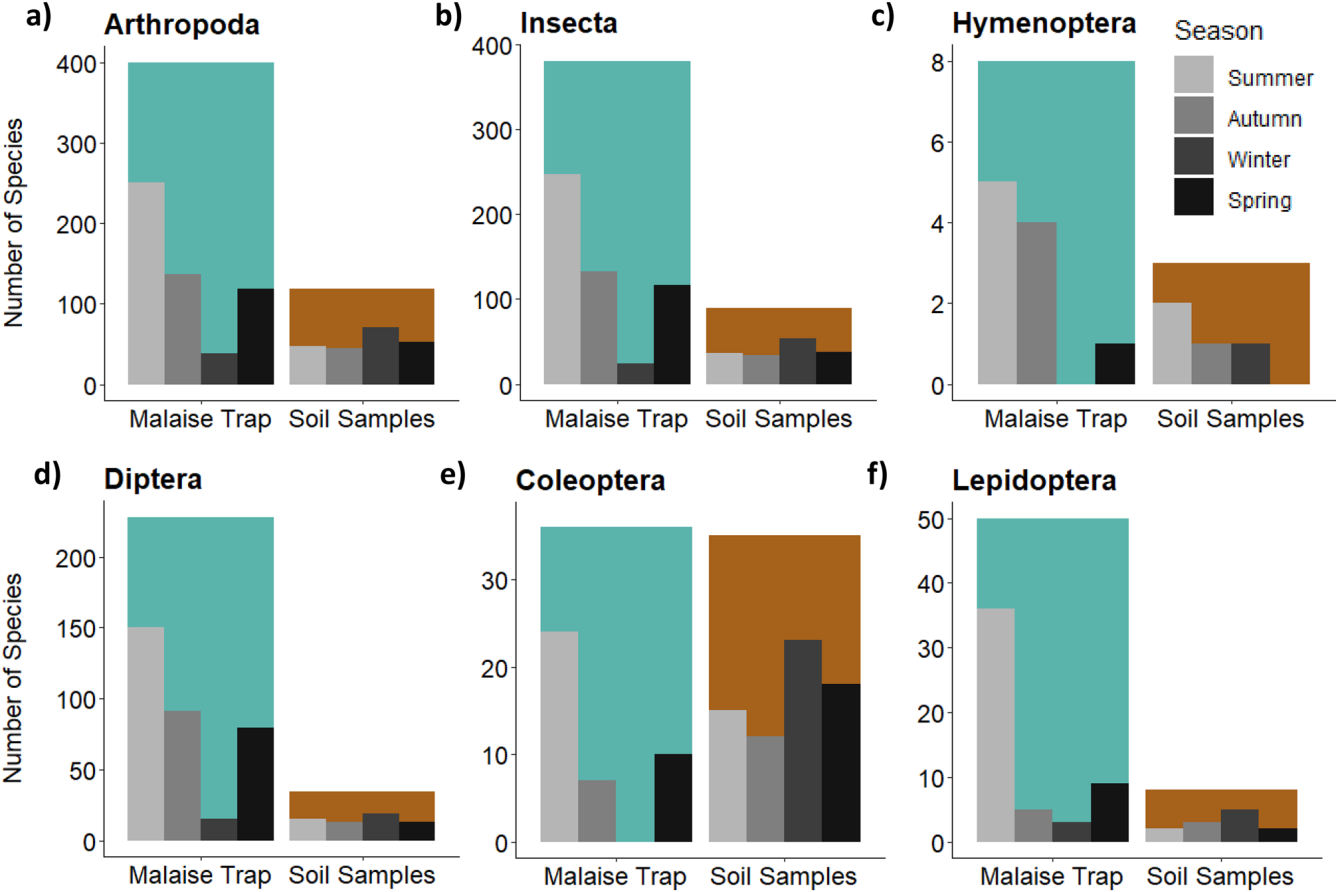
Number of arthropod species detected (blastID ≥ 99%) per sample type depending on sampling season. The colored bars indicate the total number of species detected in Malaise traps (blue) and soil samples (brown), irrespective of forest type and season. The grey shaded bars represent numbers of detected species in spring (lightest grey), summer (mid-grey), autumn (dark-grey) and winter (black).

Species composition assessed from the soil samples did not show significant differences between seasons (PERMANOVA: F_3_ = 1.233, *p* = 0.117) but showed differences between forest types (PERMANOVA: F_3_ = 2.086, *p* < 0.001). For the Malaise trap samples, a significant shift in community composition was observed across seasons (PERMANOVA: F_3_ = 17.231, *p* < 0.001) as well as between forest types (PERMANOVA: F_3_ = 3.683, *p* < 0.001) and an interaction between the two factors was also significant (PERMANOVA: F_9_ = 2.206, *p* < 0.001). The overall number of arthropod species found in the Malaise traps exceeded ca. 5.4-fold the number of species found in the soil samples (Fig. 2a). Depending on the sampling season, this proportion shifted towards the soil samples. For the Malaise trap samples, the highest total number of species was found in the summer (251 species), followed by autumn (137 species) and spring (118 species). A decrease was observed in winter, with 38 arthropod species recovered (Fig. 2a). In contrast, the number of arthropod species identified in the soil samples differed only slightly between seasons. The lowest number of arthropod species detected was 45 species in autumn, followed by summer (47 species), spring (52 species) and winter (75 species) (Fig. 2a). Similar findings were observed for the arthropod class Insecta (Fig. 2b) and the insect orders Diptera (Fig. 2d) and Lepidoptera (Fig. 2f). Over the 1-year period, only three hymenopteran species were detected in the soil samples (Fig. 2c), two during the summer and one species each in autumn and winter and none in spring. In the Malaise trap catches hymenopterans showed a peak in summer (5 species) and autumn (4 species). In winter, no Hymenoptera were observed in the Malaise bulk samples. In the summer, the total number of Coleoptera species found in the Malaise traps (24 species) exceeded the number in the soil (15 species), whereas the total number of detected species was highest in the soil samples in autumn (12 species), winter (23 species) and spring (18 species) (Fig. 2e). The highest number of Coleoptera recovered from the soil samples (23 species) was in winter, when not a single specimen was caught with the Malaise traps (Fig. 2e).

**Figure 3:**
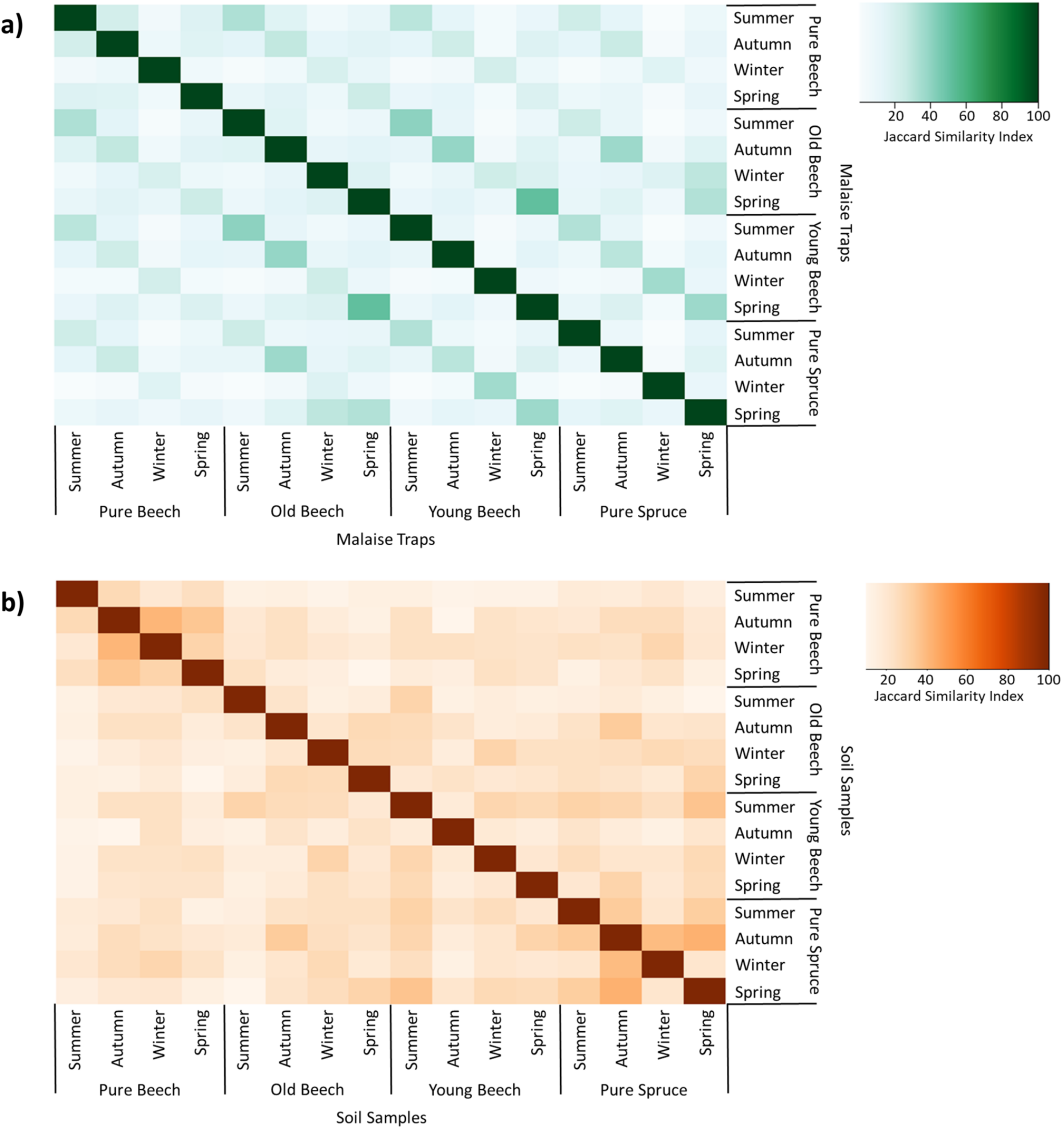
Heatmap showing similarity levels (Jaccard similarity index) between forest types on different sampling seasons. The Jaccard index was measured based on a presence-absence arthropod species matrix for a) soil samples and b) Malaise traps. Increasing similarities denoted by darker color.

For the soil samples, the highest similarity were found within the two monocultures between seasons, with an average of 27.27% and 29.13% for the pure beech and pure spruce sites, respectively (Fig. 3b). Here, arthropod communities of the two monocultures showed the highest Jaccard-similarity between autumn and winter (pure beech: J = 33.33%; pure spruce: J = 36.67%) and autumn and spring (pure beech: J = 39.29%; pure spruce: J = 40.63%). Arthropod communities from the Malaise traps showed higher similarity levels when samples were taken at the same time of year but at different locations (Fig. 3a). High similarity indices were observed between old beech and young beech stands, especially in summer (J = 41.04%) and spring (J = 51.72%), while similarities between pure beech and pure spruce were lower (summer: J = 23.74%; spring: J = 12.24%).

**Figure 4:**
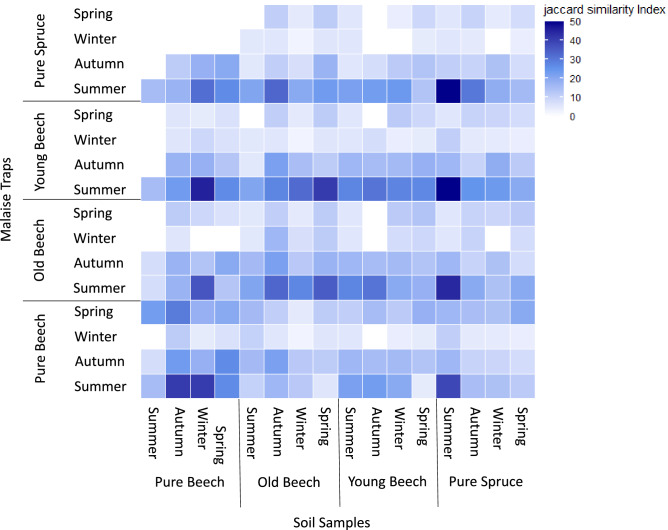
Heatmap showing similarity levels (Jaccard similarity index) between arthropod communities (blastID ≥ 99%) associated with each of the four forest types, for each sample type and season. Increasing similarities denoted by darker color.

To further analyze levels of dissimilarity between arthropod species retrieved from Malaise traps and soil samples depending on season and forest type, we used blast matches to Arthropoda with a sequence identity of at least 99% (Fig. 4). To isolate the effect of season and sample type, we observed that within all forest types, the highest community similarities were found between all soil samples (combined seasons) and the Malaise traps during summer (${\overline{\text{x}}}$ = 24.39%). For the remaining seasons the Malaise traps showed on average a lower Jaccard similarity index to the combined soil samples (autumn: ${\overline{\text{x}}}$ = 13.80%; winter: ${\overline{\text{x}}}$ = 5.40%; spring: ${\overline{\text{x}}}$ = 16.06%). To isolate the effect of season, sample, and forest type, we observed that for soil samples taken at the pure beech sampling sites, the highest similarity levels to Malaise traps (combined seasons) was when soil samples were collected in winter (${\overline{\text{x}}}$ = 16.76%). In contrast, arthropod communities from soil samples taken at the old beech sites were more similar to Malaise traps when taken in autumn (${\overline{\text{x}}}$ = 16.67%) (Fig. 4). On the other hand, soil samples of the pure spruce and young beech sites showed the highest overlap with Malaise traps when soil sampling took place in summer (${\overline{\text{x}}}$ = 19.45%; ${\overline{\text{x}}}$ = 12.85%). Overall, we observed an accumulation of species inhabiting the above ground layer during summer and in the ground layer of the pure beech sites in winter.

**Figure 5:**
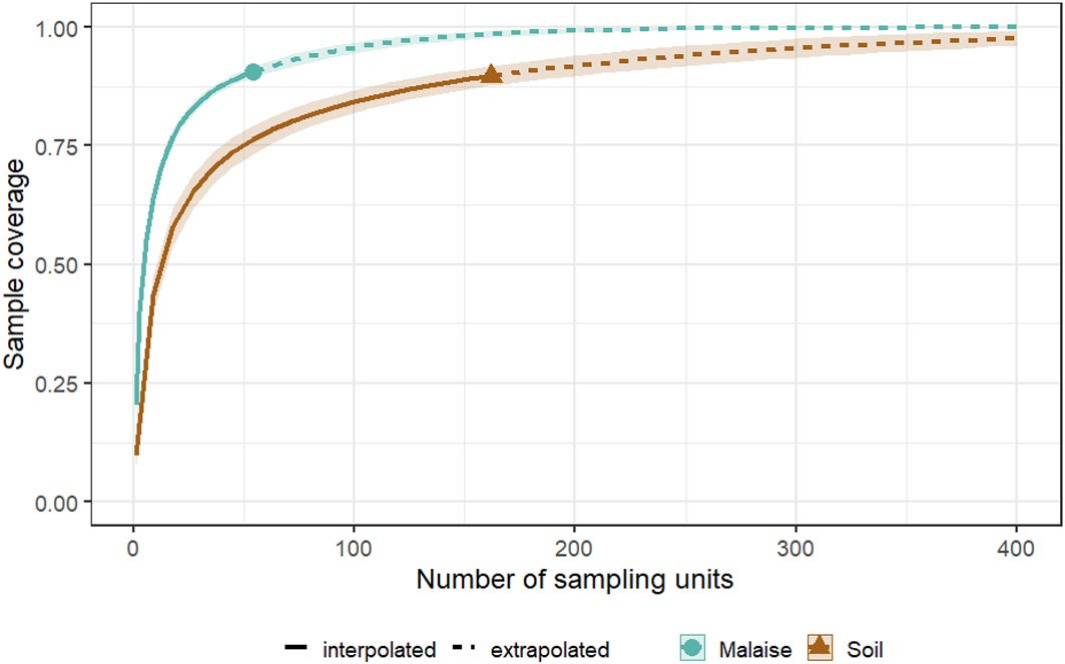
Sample completeness curves showing diversity estimates with respect to sample coverage for the different sample types, Malaise trap (green) and soil samples (brown).

The sample completeness curve showed that more soil samples than Malaise trap samples would be needed to assess total existing arthropod diversity (Fig. 5).

## Discussion

Our study on environmental DNA of forest ecosystems showed that above and below ground habitats can be complementary to detect species’ diversity patterns . Such approaches are decisive in providing temporal information on presence-absence of a species in a habitat, which in turn could facilitate the implementation of biomonitoring^19,20^. Although soil invertebrate metabarcoding from eDNA is still to mature, the species overlap observed here between Malaise traps and soil samples highlights the potential for future biodiversity assessment studies and resulting conservation strategies. Here we showed that the metabarcoding of different sample types can considerably increase the number of species detected in an ecosystem. While, as expected, flying insects were significantly more diverse in the Malaise traps, several groups of ground dwelling arthropods like Malacostraca, Chilopoda and Diplopoda were exclusively found in the soil samples. This suggests that eDNA extracted from the soil samples originated to a large degree from species that directly interact with the ground layers. This close link between species detection probability and association with the habitat was demonstrated by Arachnida. All spider species recovered from the soil samples are typical inhabitants of the litter layer or the soil itself. With the exception of *A. accentuata,* all species detected in the Malaise traps are net-building species associated with the shrub layer. Although the 14 Staphylinidae species detected in the soil samples have wings, only four were also detected in the Malaise trap catches. The ten species exclusively recovered from the soil samples are all members of ground dwelling genera^21,22^.

Although flying insects are more likely to be detected within the Malaise trap catches, four dipteran species were exclusively found in the soil samples, possibly as eggs or larvae. The reasons why these four dipteran species were absent from the Malaise traps could be manifold. As well as known methodological issues such as primer and biomass biases^23–25^ a total of 8 weeks of Malaise trapping only allows for a short glimpse of the existing flying arthropod diversity, and species with a short flight period are especially prone to be missed. For example, flying adults of two lepidopterans *Nematopogon robertella* and *Nemophora congruella* are usually observed in Germany between May and June (gbif.org), an interval during which Malaise sampling was not conducted.

As well as an association of species with the habitat, the time of sampling directly influenced species detection rates. Due to the poikilothermic character of arthropods^1^ but also due to fluctuations in food availability^26^, there is a wide consensus that arthropod and in particular insect diversity is directly linked to time of year. In this study, we observed different seasonal dynamics in arthropod communities collected from the soil and by Malaise traps. While species diversity in Malaise traps was higher during summer, species diversity associated with the soil habitat increased in the colder seasons. Flying arthropods, e.g. dipterans, hymenopterans and lepidopterans are especially active during the warm summer months^1^, which was mirrored by the high number of arthropod species found in the Malaise trap catches during the summer season. Due to decreasing temperatures and low light levels of the winter months, many insects enter a hibernation state in late autumn. While some insects prefer the bark of trees, dead wood, hedges and meadows for hibernation, many others hibernate in leaf litter or dig soil chambers. As a result, an accumulation of arthropod species was observed in soil samples during winter, highlighting the importance of soil habitats for arthropod conservation strategies.

Additionally, it must be kept in mind that in contrast to Malaise traps eDNA can persist in the soil for several weeks, and the metabarcoding of soil eDNA will allow the detection of eggs, larvae and organisms that might have already entered a state of inactivity at the time of sampling.

Arthropod community composition associated with the ground layer was more driven by forest type, while communities associated with the above ground habitat showed significant differences also between seasons. We also observed that species assessed with Malaise traps were more likely to be found in several habitats within one season. This is likely due to the fact that flying organisms range wider while ground dwelling organisms are more sessile. The sample completeness curves support these assumptions, where the above ground arthropod diversity using Malaise traps requires a comparatively lower sampling effort than the assessment of the ground arthropod diversity based on eDNA extracted from soil. However, the species lists presented here are almost certainly incomplete, as indicated by the sample completeness curves. Assuming that most biodiversity studies represent snapshots of local diversity and they very rarely reach a plateau, either due to low sampling effort or high diversity levels we calculated that ca. 200 Malaise samples and 400 soil samples would be needed to truly reflect the existing arthropod alpha diversity.

Similarly, arachnid diversity associated with temperate forests is more diverse than presented here^27,28^ and this is especially true for soil environments^29^. When the conditions are suitable, up to hundreds of thousands of mites can normally be found per square meter^29^, while we did not detect any species of this order in the soil samples. The number of hymenopterans found in the soil samples did not exceed three species. Even if approximately 70% of all described bee and wasp species nest in the ground^30^, this might not necessarily apply to forest dwelling hymenopteran species^30^. In addition, other factors to be taken into account are the small sizes of the samples analysed (100 g of soil approximately), incomplete databases as well as the fact that the hymenopterans are one of the most difficult arthropod groups to target with metabarcoding using universal primers^23^. Former studies showed that hymenopteran sequences tend to have a lower affinity to universal COI primers compared to other arthropod groups^23^. In this study, only one COI primer pair was used which could have impaired the amplification of problematic arthropod groups such as Hymenoptera but also the Acari and Collembola due to primer bias and the use of target taxon- specific primers could have circumvented this^31^.

The European web-spinning larch sawfly, *Cephalcia lariciphila* was found in soil samples taken at the pure spruce sites in autumn. The sawfly species is usually found in coniferous forests where the larva feeds on the needles of the trees. We would have expected to find the species at different times of the year as the larvae have been shown to remain in the soil for several years but females can reach the canopies by climbing up the trunk, rather than flying^32^. The short flight period combined with a rather short sampling period can be expected to be the reason why no specimens of *C. lariciphila* were found in the Malaise traps. Other methodological biases could be made accountable for this, such as DNA extraction efficiency, taxon-specific amplification bias and insufficient samples. Despite eDNA metabarcoding enabling a rapid assessment of biodiversity, it is known to have species detection errors that may occur during field sampling, molecular work and bioinformatics steps^33^. In preliminary experiments we observed that out of 26 morphologically identified Coleoptera families found in a total of six Malaise trap bulk samples, nine families were not recovered with metabarcoding. In addition, DNA extraction of the bulk samples was performed in a non-destructive manner, by overnight incubation in lysis buffer. The effectiveness of the lysis buffer on different insect groups has not been fully tested yet, but it has been shown in a recent study that highly sclerotized insects release less DNA into the preservative ethanol than soft tissued ones^16^. Less beetle DNA released could have led to false negative results, as the percentage of DNA with which a species contributes to the DNA mixture determines whether a species is recovered or not^25^.

Life-history traits have been used as a tool for biomonitoring in freshwater studies^34^. Such an approach could link biotic responses to environmental conditions, taking into account seasonal effects and habitat type. For example, in our study, the beech moth *Cydia fagiglandana* (Lepidoptera: Tortricidae) was found in the pure beech forests in the Malaise traps in summer and in the soil in autumn and winter. Although *C. fagiglandana* completes a single generation in a year, there are several stages that could remain undetected in biodiversity assessments. Adults emerge from late May to the end of October but egg-laying inside the new shoot leaves of Fagacae begins in late June to late October. In autumn the larvae drop together with the leaves and fruits to the ground where they hibernate^35^. Acknowledging for such specific life history properties (traits) provides an understanding of community structure and diversity but also serves as a powerful tool for prediction^36^. Similarly, we found the large yellow underwing, *Noctua pronuba*, a moth of the family Noctuidae (Lepidoptera: Noctuidae), in the old beech forests in the Malaise traps in summer and in the soil during winter. In this case, adult moths could be observed throughout the summer. For this species, eggs are also laid in large batches on plant leaves and fall to the ground with the leaves during autumn where the larvae finally pupate^5^. In both cases, the use of different sampling strategies and seasonal time series allowed us to find forest arthropod diversity patterns that would only be possible using a trait-based ecological approach.

Despite some methodological limitations, we showed that the type of samples metabarcoded (soil versus Malaise) significantly influenced the arthropod diversity levels observed. These results are in accordance with other studies which show strong inconsistencies in the species lists obtained between various sample sources in the same locations^16,18,37–39^. Further to this, we observed a time lagged turnover between the two sample types, which highlights that both (and eventually additional) sample types are needed when aiming to assess total forest arthropod diversity. In fact, in combination with time series, they allowed us to unravel the complex life cycles of arthropods by monitoring species turnover between forest habitats. Here we have shown that a sampling strategy encompassing time-series and different sample types can provide valuable information on the ecological dynamics and life cycles of arthropods as well as potential season induced phenological changes, in the context of long-term monitoring programs.

## Material and methods

### Sampling strategy

All sampling sites were located in the Eifel National park, situated in the south-western part of Germany close to the Belgian border (Supplementary Fig. 1, Supplementary Table 1).

In this study the sampling site comprised a forest conversion gradient from a Norway Spruce (*Picea abies*) monoculture to a European Beech forest (*Fagus sylvatica*). To reflect the different stages of conversion from spruce to beech, four forest types were defined: pure beech (PB), old beech (OB), young beech (YB) and pure spruce (PS) (Supplementary Table 1, Supplementary Fig. 2).

The forest types differed in tree species composition and tree age. The pure beech and pure spruce forest types were monoculture stands. The pure beech stands were approximately 180 years old and partly under special protection through North-Rhine Westphalia (Naturwaldzelle) (Sampling site 01). The spruce monocultures were substantially younger with ca. 60 years old. Spruces of the same age dominated the young beech sampling sites that had only recently been underplanted with beeches. At the old beech sampling sites, beeches had already reached a height of more than 3 m and actions to remove spruces from the forest were conducted.

A total of 12 Townsend Malaise traps (three per forest type) were set up in the Eifel National Park, North-Rhine Westphalia, Germany, during July 2016. To ensure that the orientation of the Malaise traps was consistent and to minimize potential biases caused by wind direction and position of sun, the highest point of each trap was set facing south. The traps were left in the field for the full duration of the experiment until April 2017, ensuring that insects were collected from exactly the same locations. In October 2016, two additional traps (Malaise Trap 13, pure spruce and Malaise Trap 14, old beech) were installed at two further sampling sites (Sample Site 13 and Sample Site 14). All traps were equipped with a bottle filled with approximately one litre of absolute ethanol (99,96%) over a 2-week period in July 2016 (13.07–27.07), October 2016 (13.10–27.10), January 2017 (11.01–25.01) and April 2017 (12.04–26.04) (Supplementary Table 2). The ethanol was replaced every week to ensure that the concentration of the preservative ethanol was stable and to avoid loss of insects a mesh filter was used (MICROFIL V Filter White Gridded 0.45 µm-diameter 47 mm & 100 ml Funnel Sterilized). Due to heavy snow during the winter period, new traps were set at the start of the new sampling season in January 2017.

Three soil samples were collected around each Malaise trap, from the organic horizon of the top 10 cm layer (excluding the litter layer). Soil sample sites were located 4 m and 5 m away from the trap, forming a triangle in the centre of which the Malaise trap was located (Supplementary Fig. 3). One corner of the sampling triangle was pointing south, while both remaining corners were pointing north west and north east, respectively.

Each sampling site was sampled four times in the course of a 1-year period. Soil sampling and Malaise trapping were synchronized and soil sampling was done on day 14 of each Malaise trapping period, when the last bottles were collected (Supplementary Table 3). Each soil sample consisted of approximately twenty 44 mm × 100 mm cores, taken 5 cm apart. A total of 162 soil samples were collected and stored at − 20 °C until further processing.

### DNA extraction

#### Bulk samples from Malaise traps

Non-destructive DNA extraction was performed by overnight incubation in lysis buffer, using a modified protocol of Aljanabi and Martinez (1997). The arthropods were sieved from the collecting ethanol using a mesh filter (MICROFIL V Filter White Gridded 0.45 µm-diameter 47 mm and 100 ml Funnel Sterilized), which was processed with the specimens. The insects were dried for 10 min at room temperature. Depending on biomass, between 15 and 25 ml of extraction buffer (0.4 M NaCl, 10 mM Tris-HCl pH 8.0, 2 mM EDTA pH 8.0) and 2% Sodium dodecyl sulphate (SDS) were added to each bulk sample. Finally, 400 µg Proteinase K was added per ml of lysis buffer and samples were lysed overnight at 52 °C on an orbital shaker at 30 rpm. The next day, the lysate was poured out of the bottles using the MICROFIL V Filter (White Gridded 0.45 µm-Dia 47 mm and 100 ml Funnel Sterilized) and a 6 M NaCl solution was added to the lysate to a concentration of 4 mmol. The samples were vortexed for 30 s, centrifuged at 4700 rpm for 30 s and the supernatant was transferred to a falcon tube and an equal volume of isopropanol was added. After careful mixing by inversion, the tubes were left at − 20 °C for 1 h and subsequently centrifuged at 4700 rpm for 60 min. The supernatant was discarded and the resulting pellet was washed with 20 ml of ice cold 70% ethanol, by centrifuging at 4700 rpm for 15 min. The remaining ethanol was discarded and the pellet was left to dry at 20 °C overnight. The pellet was then resuspended in 1 ml of sterile H_2_O and stored at − 20 °C until further processing.

#### Soil samples

DNA extraction from the soil samples was conducted using two different extraction methods: a commercial (lysis-based) DNA extraction kit (Macherey-Nagel NucleoSpin Soil) and a (no lysis) phosphate buffer protocol from Taberlet et al. 2012^40^. Each of the triplicated samples were processed individually. After defrosting the soil overnight at 4 °C, the samples were thoroughly mixed, DNA was extracted from 0.5 g of soil per sample using the Macherey-Nagel NucleoSpin Soil Kit following the manufacturer's protocol.

The second DNA extraction method allowed extracellular DNA to be extracted from larger amounts of starting material using a phosphate buffer and did not include a lysis step. Each of the three samples taken per sample site and season were treated individually. Soil samples were removed from the − 20 °C chamber approximately 12 h before DNA extraction and stored at   4 °C overnight. The next morning, each sample was thoroughly mixed and an equal weight of saturated phosphate buffer solution (Na_2_HPO_4_; 0.12 M; pH 8)^40^ was added. Samples were placed in an orbital shaker at 120 rpm for 15 min. Thereafter, duplicates were processed, where two 2 ml Eppendorf tubes were filled with 1.7 ml of the resulting mixture and centrifuged for 10 min at 10,000 g. Four hundred microliters of the resulting supernatant were transferred to a new 2 ml collection tube and 200 μl of SB binding buffer from the Macherey-Nagel NucleoSpin Soil Kit was added. Duplicate lysates were merged by loading onto a single NucleoSpin Soil Column and centrifuged at 10,000 g for 1 min. From this step onwards, the standard manufacturer's protocol for the Macherey-Nagel NucleoSpin Kit was followed from step 8. DNA was eluted with 50 μl of SE Buffer (Macherey-Nagel). Ten microliters of the resulting DNA eluate was diluted in 90 μl of pure H_2_O (Sigma), followed by DNA purification using the PowerClean Pro DNA Clean-Up Kit (MO Bio Laboratories, Inc.) following the manufacturer's protocol. For the purposes of this study, results from the two types of soil extraction were merged.

### Choice of primers and amplicon library preparation

For amplicon library preparation a primer pair targeting the 313 bp ‘mini barcode’ region of the mitochondrial Cytochrome c Oxidase subunit I gene (COI) was used^41^. The ‘mini barcode’ primer pair consisted of the forward primer mlCOIintF *5*′*-*ACACTCTTTCCCTACACGACGCTCTTCCGATCT**GGWACWGGWTGAACWGTWTAYCCYCC** *-3*′^41^ and the reverse primer dgHCO2198 *5*′*-* GTGACTGGAGTTCAGACGTGTGCTCTTCCGATCT**TAAACTTCAGGGTGACCAAARAAYCA-3**′^41^ (Illumina overhang in regular font and primer in bold). Library preparation was carried out using a two-step PCR approach^42,43^ , whereby PCR1 amplifies the gene region of interest and PCR2 adds the sample index together with the Illumina overhang (indexed primers). For each sample, a unique combination of indexes was chosen.

DNA extracts were quantified using the Quantus Fluorometer (Promega). Ten nanograms of template DNA was used for PCR1. PCR1 consisted of 7.5 µl Q5 Hot Start High-Fidelity 2 × Master Mix (New England BioLabs), 1 μl Sigma H2O, 0.5 µl forward Primer (10 µM), 0.5 µl reverse primer (10 µM), 0.5 μl Bovine Serum Albumin (Thermo Scientific) and 1 µl template DNA, making up a total of 15 µl. PCR1 cycling conditions were as follows: 2 min at 98 °C (1 ×); 40 s at 98 °C, 40 s at 45 °C, 30 s at 72 °C (20 ×); 3 min at 72 °C (1 ×). PCR products were purified with 4 µl of HT ExoSAP-IT (Applied Biosystems) to each sample, following the manufacturers’ protocol. For PCR2 (index PCR) the purified PCR products were split into two PCR tubes.

For PCR2, each tube contained 12.5 µl Q5 Hot Start High-Fidelity 2X Master Mix (New England BioLabs), 3 µl Sigma H2O, 1.2 µl of index forward primer (10 µM) (AATGATACGGCGACCACCGAGATCTACAC NNNNNNNN ACACTCTTTCCCTACACGACGC TC), 1.2 µl of index reverse primer (10 µM) CAAGCAGAAGACGGCATACGAGAT NNNNNNNN GTGACTGGAGTTCAGACGTGTGCTC) and 8 µl of purified PCR1 product. PCR2 cycling conditions were as follows: 2 min at 98 °C (1 ×); 40 s at 98 °C, 30 s at 55 °C, 30 s at 72 °C (20 ×); 3 min at 72 °C (1 ×). All tagged PCR products were visualised by gel electrophoresis and PCR bands with the expected size were excised and purified using the QIAquick Gel Extraction Kit (Qiagen). Purified PCR products were quantified using the Quantus Fluorometer (Promega) and pooled in equal concentrations. The resulting purified amplicon library pool (3 ng/µl) was sequenced on an Illumina MiSeq (MiSeq Reagent Kit v3, 2 × 300 bp) sequencing platform at Liverpool University’s Centre for Genomic Research (Liverpool, UK). Raw sequence data were deposited in the GenBank short read archive (SRA) under accession numberPRJNA681091 and PRJNA706915.

### Bioinformatics and data analysis

The raw fastq files were trimmed for the presence of Illumina adapter sequences using Cutadapt version 1.2.1. at the Centre for Genomic Research (Liverpool, UK). Sequences were trimmed using Sickle version 1.200 with a minimum window quality score of 20 and reads shorter than 20 bp were removed after trimming.

The fastq sequences were then checked for the presence of the COI primers with Cutadapt version 1.18^44^ using the following settings: maximum error rate (-e): 0.1, minimum overlap (-O): 20, minimum sequence length (-m): 50. Sequences lacking either the forward or reverse primer were removed and primer pairs were trimmed off from the remaining sequences. Subsequently, paired-end reads were merged with vsearch version 2.7.0^45^. Merged sequences with a length of 293–333 bp were retained for further analysis and filtered with a maxEE threshold of 1.0 using vsearch (version 2.7.0)^45^ before demultiplexing the fastq sequences using the script split_libraries_fastq.py implemented in QIIME1^46^ using a phred quality threshold of 19. Dereplication, size sorting, denovo chimera detection as well as Operational Taxonomic Unit (OTU) clustering with a 97% cutoff was conducted with vsearch 2.7.0^45^. Finally, an OTU table was built by using the –usearch_global function in vsearch 2.7.0^45^ followed by the python script “uc2otutab.py” (https://drive5.com/python/uc2otutab_py.html). For taxonomy assignment, representative sequences were blasted against the GBOL database (https://www.bolgermany.de/gbol1/identifications downloaded on 2nd of July 2019) using blastn 2.9.0+^47^.

The resulting OTU table was curated with LULU^48^. Curation started with an initial blasting of OTU representative sequences against each other using blastn (version 2.9.0). The following parameter settings were chosen: 'query coverage high-scoring sequence pair percent' (-qcov_hsp_perc) was set to 80, meaning that a sequence was reported as a match when 80% of the query formed an alignment with an entry of the reference file. Secondly, minimum percent identity (-perc_identity) was set to 84, requiring the reference and query sequence to match by at least 84% to be reported as a match. The format of the output file was customized using the –outfmt settings ‘6 qseqid sseqid pident’. The output file included the name of the query sequence and the name of the reference sequence next to the percentage match. The resulting OTU match list was uploaded into R (version 3.5)^49^ and the R-package ‘lulu’ (version 0.1.0)^48^ was used to perform post clustering curation using standard settings. The LULU algorithm filters the dataset for artificial OTUs and these were either classified as “daughter OTU” and merged with the corresponding “parent OTU” or were discarded from the dataset.

The resulting curated OTU table was loaded into Excel where data was formatted to upload into R (R studio running R version 3.5). Only OTUs with an assignment at species level (blastID ≥ 99%) were used for subsequent analysis. Furthermore, results from the two types of soil extraction were merged.

UpsetR plots were prepared using the R package UpSetR (version 1.4.0)^50^ for visualization of shared arthropod OTUs between sample types in each season. Differences in number of OTU proportions are shown in a Marioko plot prepared with the R package ggplot2^51^. To analyze dissimilarities between communities depending on season and sample type, Permutational Multivariate Analysis of Variance (PERMANOVA) using Jaccard distance matrices for incidence data of detected arthropod species (blastID ≥ 99%) were performed using dplyr (version 0.8.3)^52^, betapart (version 1.5.1)^53^ and vegan (version 2.5–6)^54^. In order to analyse dissimilarity differences in arthropod community composition between the different forests and seasons the Jaccard similarity index (J) was used on a presence-absence matrix based on arthropod species. Calculated Jaccard indices were visualized on a heatmap using the R package ggplot2^51^. Sample completeness curves and sample-size-based R/E curve with extrapolations of Hill numbers for incidence data based on the combined dataset for all forests and seasons were prepared using the R-package iNEXT^55^ at default settings (40 knots, 95% confidence intervals generated by the bootstrap procedure (50 bootstraps)).

To correlate community structure and diversity levels with the different seasons and forest types a Permutational Multivariate Analysis of Variance (PERMANOVA) based on the Jaccard similarity index for a presence-absence matrix of detected arthropod species (blastID ≥ 99%) was performed with the adonis function in R. Differences in arthropod community composition between the different forests and seasons was assessed using the Jaccard similarity index (J), where the higher the index, the more similar the communities.

## Supplementary Information

Supplementary Information.

## Data Availability

NCBI’s SRA database under accession number PRJNA706915 and PRJNA681091.
