# Supplementary material for: Metabarcoding Malaise traps and soil eDNA reveals seasonal and local arthropod diversity shifts
Source: Sci Rep. 2021 May 18;11:10498. doi: 10.1038/s41598-021-89950-6 (PMC8131643; doi:10.1038/s41598-021-89950-6)
Supplement: Supplementary file 1 — Supplementary Information. [file 41598_2021_89950_MOESM1_ESM.docx]

Arthropod diversity from the ground up: Seasonal overlap between soil and Malaise traps

Ameli Kirse*^1^, Sarah J. Bourlat^1^, Kathrin Langen^1^, Vera G. Fonseca*^1,2^

^1^ Centre for biodiversity monitoring, Zoological Research Museum Alexander Koenig, Adenauerallee 160, 53113 Bonn, Germany

^2^Centre for Environment Fisheries and Aquaculture Science (Cefas), Barrack Road, The Nothe, Weymouth, Dorset, DT4 8UB, UK

**Supplementary**

**Tables**

**Supplementary Table 1** Geographical and ecological characteristics of the 14 sampling sites. Coordinates (altitude N and latitude E) and the associated local forest type are indicated for each sampling site

| Sample Sites | Coordinates | Forest Type |
| --- | --- | --- |
| Sample Site 01 | 50° 34'11.7984''N 6°21'32.1012''E | Pure Beech |
| Sample Site 02 | 50° 34'07.7016''N 6°21'27.3996''E | Pure Beech |
| Sample Site 03 | 50° 34'12.9000''N 6°21'27.3996''E | Pure Beech |
| Sample Site 04 | 50° 32'44.5992''N 6°20'15.2988''E | Young Beech |
| Sample Site 05 | 50° 32'41.3016''N 6°20'15.6984''E | Young Beech |
| Sample Site 06 | 50° 32'29.7996''N 6°20'11.1012''E | Young Beech |
| Sample Site 07 | 50° 32'29.7996''N 6°20'11.1012''E | Old Beech |
| Sample Site 08 | 50° 31'35.1984''N 6°20'25.2996'E | Old Beech |
| Sample Site 09 | 50° 32'48.3000''N 6°20'03.4008''E | Old Beech |
| Sample Site 10 | 50° 30'17.2008''N 6°19'48.1008''E | Pure Spruce |
| Sample Site 11 | 50° 30'18.2988''N 6°19'51.4020''E | Pure Spruce |
| Sample Site 12 | 50° 33'15.8004''N 6°21'07.3008''E | Pure Spruce |
| Sample Site 13 | 50° 30'16.0056''N 6°19'51.4704''E | Pure Spruce |
| Sample Site 14 | 50° 32'49.9632''N 6°20'00.7296''E | Old Beech |

**Supplementary Table 2** Malaise trap collection periods. Time of year, number of traps and time period of collection is indicated for each sampling season.

| Season | Time of the year | Number of Traps | Sampling Dates |
| --- | --- | --- | --- |
| Season 1 | Summer | 12 | 13.07.2016 – 27.07.2016 |
| Season 2 | Autumn | 14 | 13.10.2016 – 27.10.2016 |
| Season 3 | Winter | 14 | 11.01.2017 – 25.01.2017 |
| Season 4 | Spring | 14 | 12.04.2017 – 26.04.2017 |

**Supplementary Table 3** Soil sample collection periods. Time of year, number of samples and date of collection is indicated for each sampling season.

| Season | Time of the year | Number of Samples | Sampling Dates |
| --- | --- | --- | --- |
| Season 1 | Summer | 36 | 27.07.2016 |
| Season 2 | Autumn | 48 | 27.10.2016 |
| Season 3 | Winter | 48 | 25.01.2017 |
| Season 4 | Spring | 48 | 26.04.2017 |

**Figures**


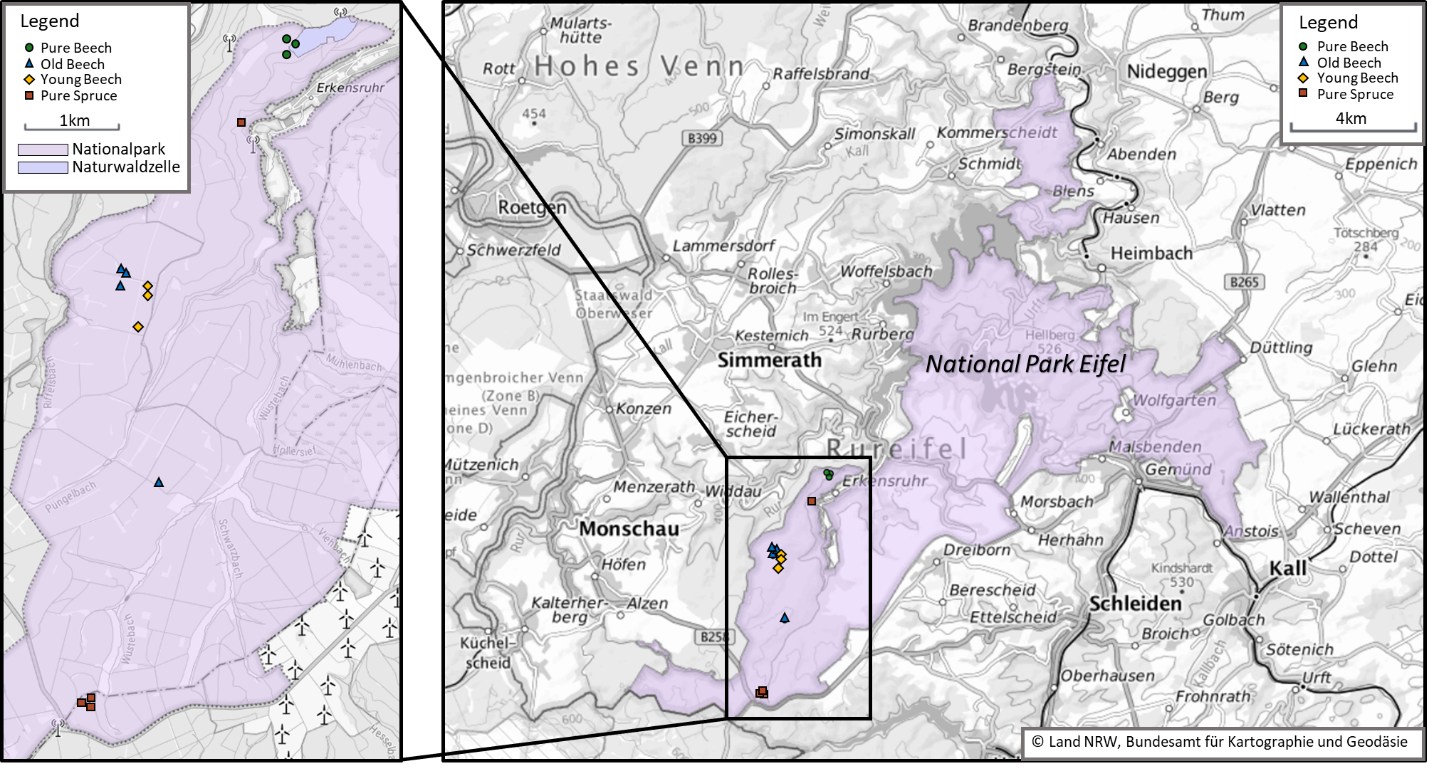


**Supplementary Figure 1** Location of the sampling sites. The area highlighted in purple corresponds to the Eifel National Park. Maps were downloaded from <https://www.geoportal.nrw/> on 20^th^ of November 2019 and modified according to our purposes using Microsoft PowerPoint 2016.


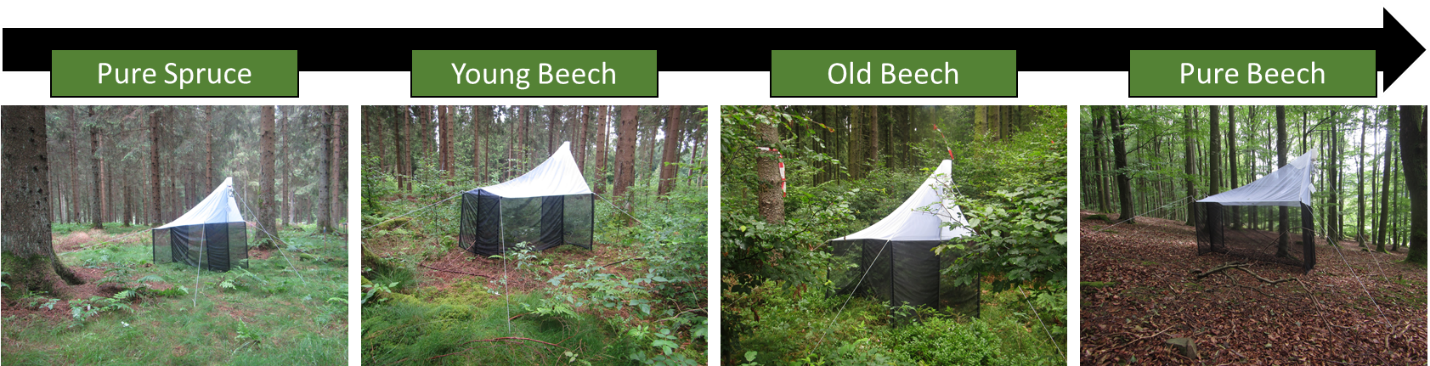


**Supplementary Figure 2** Forest conversion gradient: From pure spruce monocultures to spruce forests underplanted with beeches to beech monocultures. Photos were taken during summer (July 2016) by Ameli Kirse.


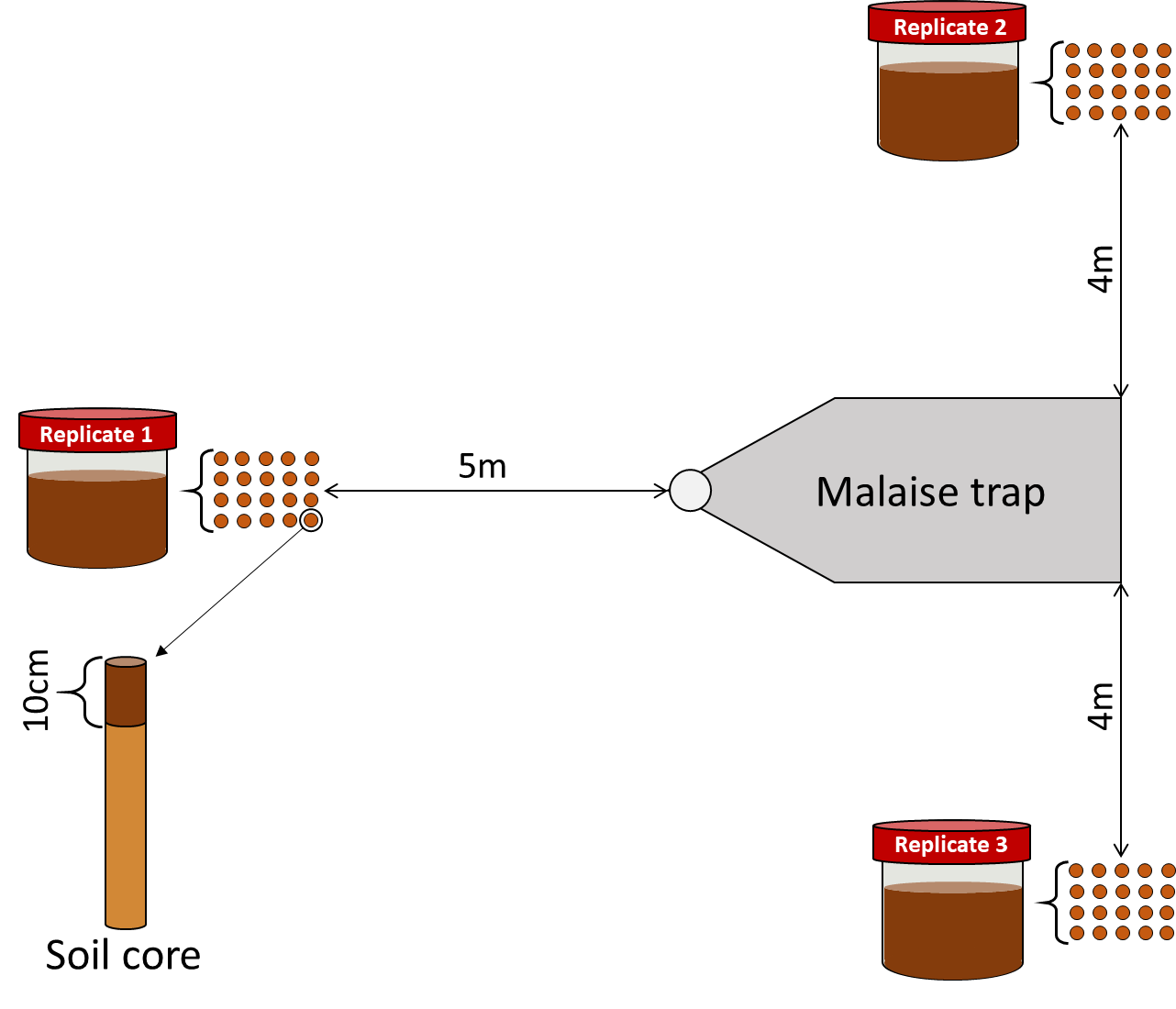


**Supplementary Figure 3** Overview of the study set up. The Malaise trap is at the centre of a triangle formed by soil sampling locations. For soil sampling the upper 10cm of soil were taken. At each sampling site three biological replicates, each consisting of 20 soil cores were taken.


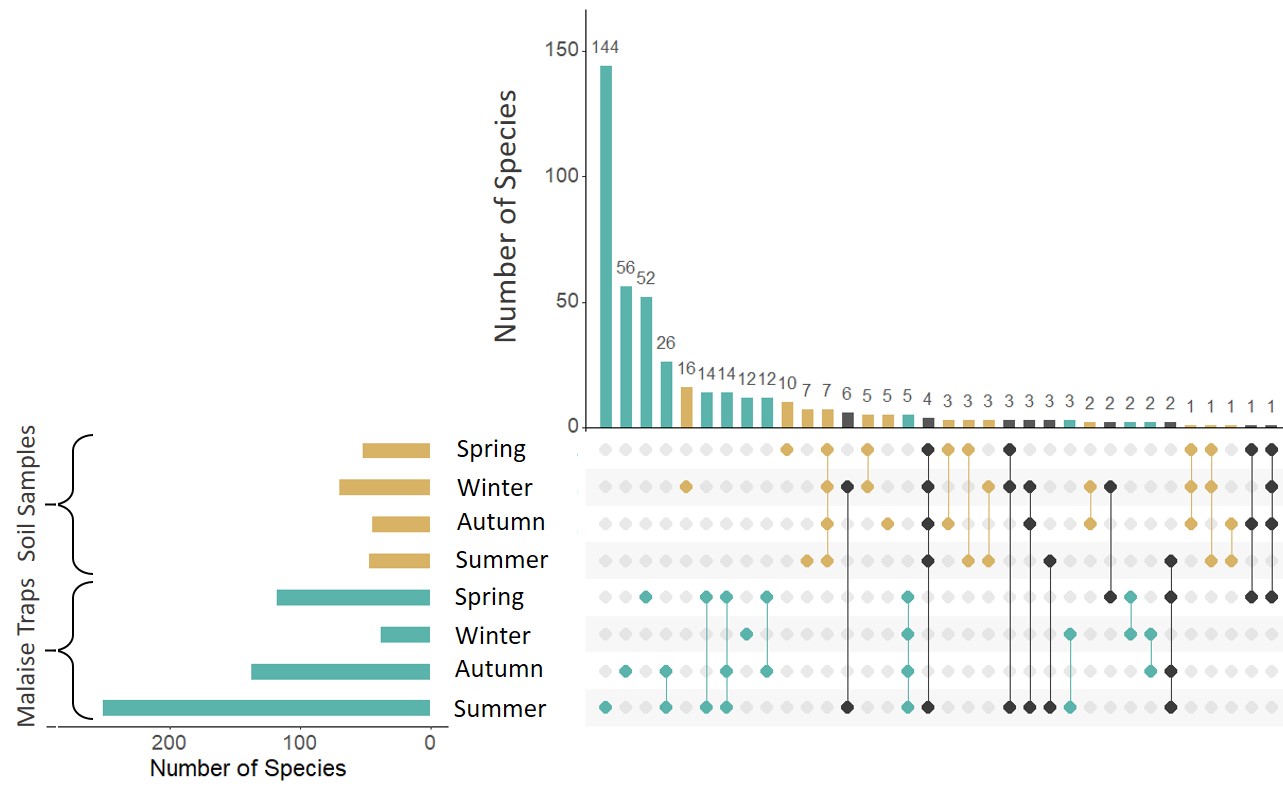


**Supplementary Figure 4** UpsetR plot showing number of unique and shared arthropod species between sample types in each season. Green-blue bars: malaise traps and yellow bars: soil samples.
